# Relative absorption of silicon from different formulations of dietary supplements: a pilot randomized, double-blind, crossover post-prandial study

**DOI:** 10.1038/s41598-021-95220-2

**Published:** 2021-08-13

**Authors:** N. Boqué, R. M. Valls, A. Pedret, F. Puiggrós, L. Arola, R. Solà

**Affiliations:** 1 Eurecat, Centre Tecnològic de Catalunya, Unitat de Nutrició I Salut, Av. de La Universitat, 43204 Reus, Spain; 2 Functional Nutrition, Oxidation and Cardiovascular Diseases Group (NFOC-Salut), Facultat de Medicina I Ciències de La Salut, Universitat Rovira I Virgili, Reus, Spain; 3 Facultat de Química, Grup de Recerca en Nutrigenòmica, Universitat Rovira I Virgili, Tarragona, Spain; 4 Hospital Universitari Sant Joan de Reus, Reus, Spain

**Keywords:** Medical research, Drug development, Clinical trial design, Drug discovery, Pharmacology

## Abstract

The purpose of the present study was to compare the relative absorption of a new powder presentation of silicon (Si) as orthosilicic acid with maltodextrin (Orgono Powder) compared to usual Si liquid presentations as orthosilicic acid with Equisetum arvense and Rosmarinus officinalis (G5 Siliplant) and orthosilicic acid with aloe vera (G7 Aloe). All dietary supplements were administered at the same Si oral dose (21.6 mg) in a randomized, double-blind, crossover post-prandial study conducted in 5 healthy men. Urine was collected at baseline and over the 6-h post-dose period in 2 separate 3-h collections for the analysis of Si concentration, which was conducted by inductively coupled plasma optical emission spectrometry as the gold standard method. No significant differences in total urinary Si excretion were found after the intake of these 3 dietary supplements; 34.6%, 32.4% and 27.2% of the ingested Si from G7 Aloe, G5 Siliplant and Orgono Powder, respectively, was excreted in urine over the 6-h follow-up period. The 3 different oral Si formulations tested, in powder and liquid presentations, provide highly bioavailable Si and present an equivalent relative absorption in healthy humans.

## Introduction

The health benefits of silicon (Si) have been widely reported over the last decades ^1^. Both in vitro and in vivo studies point out that Si exerts beneficial properties on the structural integrity of nails, hair and skin and on the synthesis of collagen and bone mineralization ^1^. Thus, oral supplemental Si is widely used in humans for improving osteoporosis ^2^, hair loss and nail and hair quality ^3^.

Si is the second most common element in the Earth’s crust behind oxygen ^4^. This mineral is naturally present in foods as Si dioxide (SiO_2_) and silicates, while most Si in water is present as free orthosilicic acid (OSA; H_4_SiO_4_) ^5^. The bioavailability of Si in foods and beverages depends on the Si total content and the form presented ^6,7^. According to the European Food Safety Authority (EFSA), the estimated average dietary intake of Si is 20–50 mg/day ^8–10^, corresponding to 0.3–0.8 mg silicon/kg body weight/day for a 60-kg person, and these intakes are unlikely to cause adverse effects ^5,6,11^. However, no recommended dietary allowance for Si has been established.

However, similar to foods, the bioavailability of Si in oral dietary supplements greatly varies depending on the chemical form. OSA, a monomeric form, is one of the most bioavailable sources of Si due to its high solubility ^12,13^. In contrast, oligomeric and polymeric forms are poorly absorbable in the gastrointestinal tract. In this sense, a study by Sripanyakorn et al. ^14^ revealed that 43% of the Si administered as OSA was absorbed, whereas the bioavailability of orally polymeric Si was less than 5%. Other works have described similar results for OSA, showing urinary excretion of approximately 50% of the ingested Si dose ^15^. Nevertheless, when OSA is present at concentrations higher than 2 mM, as in most products, it undergoes dehydration and polymerization, which leads to a reduced solubility and hence to a low bioavailability ^5^. Extensive polymerization and subsequent precipitation of OSA can be prevented by the addition of choline or other molecules as stabilizing agents, improving its absorption and thus its availability to be used or stored ^5,16^.

The absorbed Si is excreted mainly through the kidney ^15^. Because Si does not bind to any plasma proteins, it is readily filtered by the renal glomerulus, leading to high renal Si clearance. Therefore, the urinary excretion of Si is considered a good marker of the absorption of this element and an easy way to determine Si bioavailability ^15,17^, as urine samples meet three essential characteristics for obtaining biological samples in human intervention studies: a non-invasive method that is easy and rapid to obtain from subjects participating in a study. However, since faecal excretion is another route for Si elimination ^17^, urine measurements may underestimate the amount of Si absorbed.

To consolidate data on the possible beneficial effects of Si in humans, one of the strategies of the food sector has been to develop new formulations of bioavailable Si. Consequently, many forms of Si dietary supplements are found on the market, and assessing Si bioavailability is a key step to explain its effects.

In the present work, the Si dietary supplements evaluated contained OSA with maltodextrin (Orgono Powder), OSA with a mixture of plant extracts (G5 Siliplant) and OSA with aloe vera (G7 Aloe). Accordingly, the aim of the present study was to determine the relative absorption of Si from 3 different oral Si formulations by using urinary Si excretion as a measure of Si absorption.

## Methods

### Oral Silicon dietary supplements

Oral Si dietary supplements, in both liquid and powder form, were kindly provided by Silicium España Laboratorios (Vila-seca, Spain): a) G5 Siliplant, in liquid form of 60 mL, which contained 21.6 mg of OSA with a mixture of plant extracts (500 mg/L Equisetum arvense and 250 mg/L Rosmarinus officinalis) and without preservatives (batch number 15033); b) Orgono Powder, in powder form of 1.4 g, which contained 21.6 mg of OSA (providing a high amount of silicic acid in monomeric form at a concentration of 1.5% of elemental silicon and 5% of monomeric OSA) microencapsulated with maltodextrin and without preservatives (batch number OSP 1407); and c) G7 Aloe, in liquid form of 120 mL, which contained 21.6 mg of OSA with aloe vera (fresh aloe vera juice q.s. (quantum satis) 1 L Aloe barbadensis Miller), 100% organic pulp, 500 mg/L potassium sorbate and 350 mg/L citric acid) (batch number 14097). The administered doses were G5 Siliplant, 60 mL; Orgono Powder, 1.4 g; and G7 Aloe, 120 mL. Orgono Powder was dissolved in ultra-purified water to reach a final volume of 120 mL. Before product preparation, each container product was ensured to have an identical appearance. The blinded oral Si dietary supplements were sequentially numbered 111, 222, and 333, corresponding to G5 Siliplant, Organo Powder and G7 Aloe, respectively. All the dietary supplements were finally presented in the same volume (120 mL) to preserve the blindness in appearance or any other physical characteristics, and they were similar in smell and taste. Moreover, the Si supplements were administered orally in opaque glass to avoid visual interference. An independent researcher not related to the study was the person who prepared the final presentation of the Si dietary supplements guaranteeing the blindness of the participant and the researcher.

The administered Si dose was 21.6 mg for each product under strictly standardized conditions.

The Si content of the tested dietary supplements was analysed by inductively coupled plasma optical emission spectrometry (ICP-OES; PerkinElmer) at a wavelength of 251.611 nm, which is specific to silicon, with a detection limit of approximately 1 ng/ml and an accuracy of ± 5%. The results are expressed as percentages, mg/L or mg/kg. Briefly, 0.5 g of sample was weighed into Teflon glass for microwave digestion at an elevated temperature by adding 6 mL of concentrated hydrochloric acid and 2 mL of concentrated nitric acid. After digestion, the sample was dissolved in 25 mL of HPLC grade deionized water.

For the preparation of the liquid formulations (G5 Siliplant and G7 Aloe), a silanol precursor was hydrated in water, and the solution was diluted according to the concentration specifications of each product. Subsequently, other ingredients, such as preservatives and additives, were added, and finally, dietary supplements were packaged for consumption. For the elaboration of the powder formulation (Orgono Powder), a silanol precursor was hydrated in water and mixed with maltodextrin for micro-encapsulation.

Stability studies during the research and development phase of the three dietary supplements showed that the composition of the solution of silicic acid and aloe vera provided greater stability to the product. Rosmarinus has been included in the formulation to take advantage of its antioxidant and natural preservative properties.

Briefly, the stability studies consisted of 2 shelf-life studies (under normal conditions where samples were stored at 22 °C for 24 months and under accelerated conditions where samples were stored at 37 °C for 9 months) carried out with 45 bottles of 1-L capacity of the G7 Aloe product packaged in its final format. Three openings were carried out at 8, 16 and 24 months in the study under normal conditions and at 3, 6 and 9 months in the study under accelerated conditions. At each sampling point, physicochemical, microbiological and organoleptic analyses were conducted.

### Subjects

Five healthy adult men (aged 30–37 y) were recruited at the Hospital Universitari Sant Joan and EURECAT-Technological Centre of Nutrition and Health (Reus, Spain) between March 2017 and April 2017. All subjects had serum creatinine levels in a normal range (between 0.7 and 1.3 mg/dl) and provided written informed consent prior to enrolment in the trial. Exclusion criteria included the following: presented chronic diseases; had gastrointestinal disorders in the active phase; had been taking Si supplements or medicines that contain Si for 7 days before the first visit; had been participating in another clinical trial or nutritional intervention study within the last 30 days; and inability of the volunteer to follow the study guidelines.

### Sample size

To estimate the sample size, urinary excretion of Si was considered an indicator of its bioavailability. Based on the results from another study ^14^ wherein the standard deviation of the urinary excretion of Si was found to be 9.4%, it was calculated that 5 participants would be required to detect a difference of up to 20% in the urinary excretion of Si between the Si products, with 90% power at the two-sided 5% significance level.

### Study design

The study was approved by the Clinical Research Ethics Committee of the Hospital Universitari Sant Joan (15-02-26/2assN1), and all procedures and protocols were implemented in accordance with the Declaration of Helsinki and the International Conference of Harmonization and Good Clinical Practice (ICH GCP) and reported as CONSORT criteria. The trial was registered with Clinical-Trials.gov: number NCT03108508 (11/04/2017). This was a randomized, double-blind, crossover study designed to compare the relative absorption of 3 different Si supplements. The design of the study is detailed in Supplemental Figure S1. Participants who met the selection criteria were assigned a randomization code obtained from a randomization list following the chronological order in which they were included. This code indicated the sequence of consumption of the 3 products. The randomization sequence was generated using the web-based Randomizer (version 1.8.4) from the Institute for Medical Informatics, Statistics and Documentation of the Medical University of Graz and was prepared by a researcher who was not involved in the study. The randomization list remained closed until the end of the experimental intervention and once all the data had been recorded.

A total of 4 visits were scheduled for each subject: a pre-selection visit and 3 study visits with a one-week wash-out period between them ^14,15,17^. At the pre-selection visit, inclusion and exclusion criteria were checked through a medical history and physical examination and blood sampling for biochemical analyses. Subjects were asked to avoid consumption of Si-rich beverages and foods, including beer, coffee, breakfast cereals, and certain fruits and vegetables, such as bananas and green beans, during the 24 h prior to the post-prandial test. The 3 postprandial studies were performed at HUSJ-Eurecat and lasted from 08.00 am to 02:00 pm. At each study visit, the subject collected a fresh urine sample after overnight fasting. Similarly, the corresponding study product was administered orally in an opaque glass to avoid visual interferences, after overnight fasting and to avoid meal interferences. No food was consumed during the 6 h that the post-prandial study lasted. Urine was collected for the next 6 h in two fractions of 3 h (08.30–11.30 h and 11.30–14.30 h) and kept in two separate containers at -80 °C. One container was used to collect the 0–3-h fraction, and another container was used to collect the 3–6-h fraction. The urine volume of each period was measured and registered for both fractions: the 0–3-h fraction and the 3–6-h fraction. Finally, the two volumes obtained in the two urine collection fractions were added for the total volume and considered in the Si content calculation. Subjects were allowed to ingest only ultra-purified water during the study.

### Subject data collection

Baseline data included age, height, body weight, blood pressure, heart rate, smoking and drinking habits, current medication, nutraceutical and dietary supplement intake, personal and family disease history, and a complete biochemical profile. A 24-h dietary recall interview was conducted by a dietitian at each visit. Changes in medication and nutraceuticals or dietary supplements were recorded at each visit. All serious and non-serious adverse events were recorded and coded under the Medical Dictionary for Regulatory Activities (MedDra dictionary; version 19.1).

### Main outcome: silicon urine analysis

Urine samples were introduced into a volumetric flask, and a volume of yttrium solution was added as an internal standard and made up to 20 mL with ultra-pure water. Analysis of Si was carried out by inductively coupled plasma optical emission spectrometry (ICP-OES; PerkinElmer Optima model 7300 DV) with a concentric nebulizer and a cyclonic chamber after calibration. The quantification limit was 0.100 mg/L, and the detection limit was 0.033 mg/l in the digested sample, meaning 1.3 mg/l in the original sample. Total Si excretion was calculated as urine volume (l) x silicon concentration (mg/l) and expressed in milligrams.

### Statistical analyses

Data are expressed as the means ± standard deviations (SDs) or frequencies and percentages, according to the nature of the variable. Intra-individual variation in baseline urinary Si excretion between study periods was analysed by ANOVA. The effects of the different OSA formulations on urinary Si excretion were analysed by an ANCOVA model with the baseline value as a covariate and treatment, period and sequence as fixed factors, followed by the least significance difference (LSD) post hoc test to determine differences between treatments. Changes over time in urine Si levels were analysed by repeated-measures ANOVA. The significance level was set at bilateral 5%. All statistical analyses were performed with SPSS Statistics 19 (SPSS Inc., Chicago, IL, USA).

## Results

Five participants were assessed for eligibility, and 5 were randomized and therefore allocated to one of the three intervention sequences. Finally, all of the participants completed the post-prandial study.

The baseline characteristics of the participants are summarized in Table 1. The mean ± SD age was 33.4 ± 3.3 years, the BMI was 27.4 ± 1.9 kg/m^2^, and plasma creatinine levels were 0.90 ± 0.08 mg/dL. No adverse effects were reported during the study.

**Table 1:** Baseline characteristics of the study subjects.

Variable	Mean ± SD
Age; years	33.4 ± 3.3
Weight; kg	84.9 ± 11.9
Height; m	1.78 ± 0.05
BMI; kg/m^2^	27.4 ± 1.9
Systolic blood pressure; mmHg	123.4 ± 10.8
Diastolic blood pressure; mmHg	66.6 ± 3.7
Heart rate; bpm	63.2 ± 6.4
Glucose; mg/dL	92.6 ± 7.9
Creatinine; mg/dL	0.90 ± 0.08
Total bilirubin; mg/dL	0.53 ± 0.14
AST; UI/L	24.6 ± 6.8
ALT; UI/L	27.6 ± 9.3
GGT; UI/L	30.4 ± 13.3
Alkaline phosphatase; mg/dL	82.0 ± 24.3
Total cholesterol; mg/dL	187.4 ± 27.9
HDL cholesterol; mg/dL	58.2 ± 22.0
LDL cholesterol; mg/dL	104.4 ± 23.7
Triglycerides; mg/dL	124.0 ± 55.6
TSH; mcUl/mL	1.95 ± 0.76
Hemoglobin; g/dL	14.6 ± 0.5
Smoker; n (%)	1 (20%)

Values are means ± SD or frequencies and percentages, as indicated. n = 5. AST, aspartate aminotransferase.

ALT, alanine aminotransferase; GGT, gamma-glutamyl transferase, TSH, Thyroid-Stimulating Hormone; HIV, human immunodeficiency virus.

**Figure 1:**
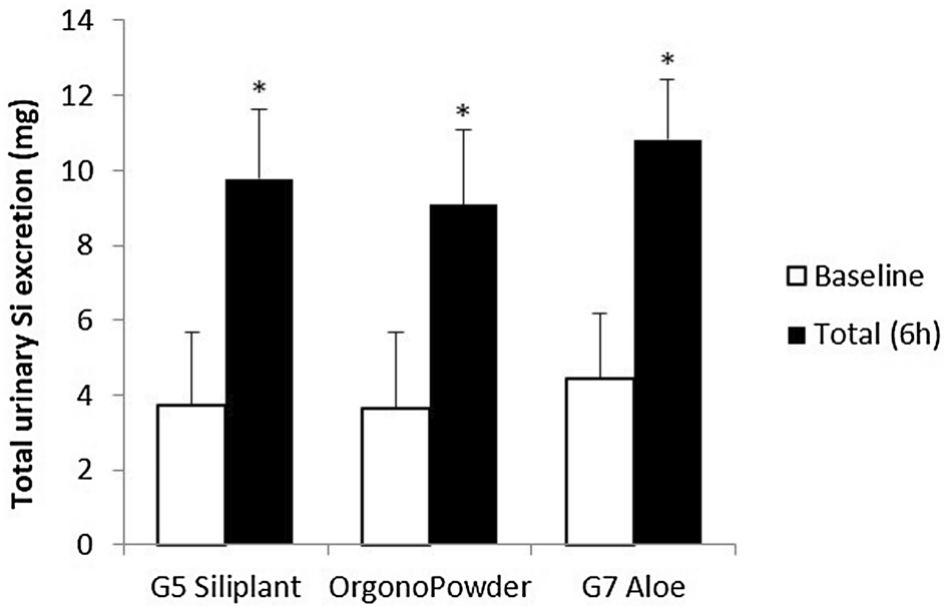
Urinary Si excretion at baseline and over 6 h after administration of G5 Siliplant (liquid presentation), Orgono Powder (powder presentation) and G7 Aloe (liquid presentation; all containing 21.6 mg of Si) in healthy subjects. The results are expressed as total milligrams excreted in urine. Values are the mean ± SD (n = 5) *, *p* < 0.05 with respect to baseline.

The basal levels of urinary Si excretion (total milligrams excreted) in fasting conditions prior to supplement administration were similar between subjects allocated to the various treatments (ANOVA, p = 0.904) (Fig. 1). In terms of concentration, the baseline Si levels in urine were 10.5 ± 3.0 mg/L before G5 Siliplant intake, 11.0 ± 3.0 mg/L before Orgono Powder intake, and 9.3 ± 2.3 mg/L before G7 Aloe intake without significant differences among them.

The ingestion of G5 Siliplant, Orgono Powder and G7 Aloe all markedly increased the total urinary excretion of Si after 6 h (Fig. 1). Therefore, G5 Siliplant induced an increase in Si excretion of 163% above baseline (p = 0.002), Orgono Powder induced an increase of 149% (p = 0.026), and G7 Aloe induced an increase of 142% (p = 0.001). Moreover, the comparison of Si excretion across the 3 dietary supplements controlling for baseline values showed no difference in the total excretion of Si 6 h after consumption (*p* = 0.238) (Fig. 1). The increase in urinary Si over the 6-h period accounted for 34.6 ± 3.11% of the administered Si dose for G7 Aloe, 32.4 ± 3.43% for G5 Siliplant and 27.2 ± 2.83% for Orgono Powder.

**Figure 2:**
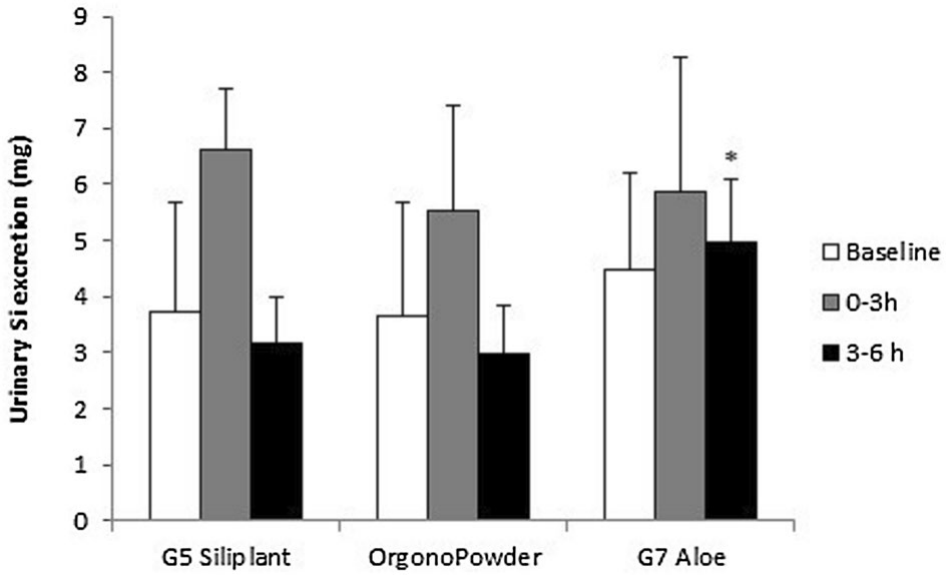
Urinary Si excretion at baseline and over the two collection time periods (0–3 h and 3–6 h) after administration of G5 Siliplant (liquid presentation), Orgono Powder (powder presentation) and G7 Aloe (liquid presentation; all containing 21.6 mg of Si) in healthy subjects. The results are expressed as total milligrams excreted in urine. Values are the mean ± SD (n = 5) *, *p* < 0.05 with respect to G5 Siliplant and Orgono Powder at the same time period.

To obtain information on ​​the kinetics of absorption and urinary excretion of the Si dietary supplements, we analysed urinary excretion in two separate time periods: 0–3 h and 3–6 h (Fig. 2). This analysis revealed that although the Si levels excreted during the first 3 h were not significantly different after the intake of the 3 different dietary supplements (p = 0.514), the amount of urinary Si excreted throughout the collection period from 3 to 6 h after G7 Aloe intake was significantly higher than after the ingestion of G5 Siliplant and Orgono Powder (*p* < 0.01).

Moreover, while Si excretion in urine over the 3–6-h interval was below baseline after supplementation with G5 Siliplant and Orgono Powder, after G7 Aloe intake, urinary Si levels remained higher than baseline in this time period (Fig. 2).

## Discussion

In the present study, we examined the relative absorption of Si provided by 3 different Si formulations in healthy humans. Our data show that all OSA dietary supplements provide highly bioavailable Si considering the significant rise in the urinary excretion of Si over a 6-h period after the ingestion of each product, without differences in urinary Si excretion between the 3 products. This suggests a similar Si absorption from these 3 dietary supplements, indicating that they have an equivalent relative absorption. Although the three supplements contain Si in the same chemical form, that is, orthosilicic acid, the presentation form (liquid versus powder) and the rest of the ingredients of the formulation could affect Si bioavailability. In this sense, the presence of other nutrients or ingredients together with a mineral, in a supplement or in the diet, could improve or diminish its bioavailability, since they can interact through binding or competitive interaction ^18^. Moreover, since it is known that solubility affects Si absorption ^19^, our results may suggest that the powder product (Orgono Powder) presents similar solubility, once dissolved in water, to the liquid formulations, and this results in a similar relative absorption. Finally, it has been reported that at higher concentrations, polymers or colloids of silica are generated, which have a lower bioavailability ^14^. However, the different initial Si concentrations of the studied products do not appear to affect Si absorption.

The dose of oral Si used in this study, 21.6 mg, was based on the current information provided by the EFSA Panel on Dietetic Products, Nutrition and Allergies, which considers and estimates a consumption of 20 to 50 mg of Si per day being unlikely to produce adverse effects ^5^. In addition, in a study on Si uptake, 21.5 mg of Si was considered the positive control ^14^.

Renal function seems to be a key factor in determining the plasma concentration of Si. Indeed, in healthy individuals, the renal clearance of plasma Si is 70–80%, with a total Si daily excretion of approximately 20 mg, while hypersilicaemia occurs in individuals with impaired renal function ^20,21^. In fact, the urinary excretion of Si is considered a reliable and accurate measure of its absorption after an overload ^17^. Moreover, the reliability of the relative absorption data obtained in urine is reinforced with the results of several studies, which demonstrated that the concentration of Si in blood correlated significantly with the levels of Si excreted in urine. Therefore, and according to the conclusions from these different studies ^15,17,22–24^, the urinary excretion of Si is recognized as an appropriate indicator of Si absorption.

After oral Si absorption, two routes of biodistribution from the gastrointestinal tract have been proposed: rapid urinary excretion for the majority of Si, approximately 90% of the absorbed Si with an approximate half-life of 2.7 h, and slow excretion for the remaining 10% derived from Si captured and metabolized by the tissues with a half-life of approximately 11.3 h ^25^. Consequently, if some Si is retained by the tissues, urinary excretion could be delayed until approximately 12 h. However, urinary Si excretion is comparable when using urine collected for 24 h or shorter fractions (0–6 or 0–8 h) ^17^. Thus, the sample collection period covered in the present study (0–6 h) is sufficient to precisely estimate the absorption of Si from the 3 products.

Accordingly, the analysis of Si bioavailability from different foods and supplements performed in a crossover study, based on urinary Si excretion in a 6-h period ^14^, showed that organic Si in the form of monomethyl silanetriol displayed the highest percentage of absorption at 63% (i.e., the percentage of the dose consumed excreted in urine), followed by a low-concentration OSA solution and choline-stabilized OSA that showed 43% and 16% absorption, respectively. In contrast, the lowest Si absorption percentages were observed for magnesium trisilicate (4%) and colloidal Si (1%) ^14^. Thus, the absorption of Si from both foods and dietary supplements depends not only on whether it is organic or inorganic but also on whether it is in monomeric or polymeric form and, in the latter case, its degree of polymerization ^14^.

In the present study, the percentage of absorption was 35% for G7 Aloe, 32% for G5 Siliplant and 27% for Orgono Powder. Therefore, we can state that the relative absorption of OSA with maltodextrin (Orgono Powder) seems to be considerably higher than the relative absorption of choline-stabilized OSA, with a percentage of absorption of 16%, and is higher than magnesium trisilicate (4%) or colloidal Si (1%) ^14^. Moreover, a Si amino acid complex and powdered horsetail grass showed a percentage of absorption of 12% over a 10-h urine collection period ^25^, while the percentage of absorption of a vanillin-OSA complex was 21% over 6 h ^16^, which was also lower than that of OSA with maltodextrin. Our results suggest that the preparation of OSA with maltodextrin could be an efficient method to maintain high OSA solubility by avoiding the polymerization of OSA into oligomers, polymers or even colloids, thus allowing us to obtain a Si product with high bioavailability.

The study of Si bioavailability is of great interest for its impact on human health. Human, animal, and in vitro studies indicate that Si in nutritional and supra nutritional amounts promotes bone and connective tissue health, may have a modulating effect on the immune ^26^ or inflammatory response, and has been associated with mental health. Moreover, epidemiological studies showed that dietary Si was favourably related to markers of bone density and turnover. Moreover, Si in nutritional amounts may lower the risk of Alzheimer’s disease and may improve photo-damaged skin or hair and nail conditions ^3,27–29^. In future studies, to determine whether Si derived from the intake of G5 Siliplant, Orgono Powder and G7 Aloe supplements has been retained in tissues or metabolized by cells, Si balance must be analysed using isotopic labelling, either by stable or radioactive isotope methods. In this sense, the results from an interesting pilot balance study ^17^ reported that in healthy subjects, almost the total ingested dose of orthosilicic acid was recovered in urine and faeces within 24 h, indicating that the same quantity of ingested Si was excreted. Although this study was not able to prove if the excreted Si came from the ingested Si or if there had been an exchange with the Si reserves of the organism, the authors argued that this may indicate that Si metabolism is regulated but that the Si reserves of these individuals were just replete. Moreover, further studies are needed to determine the bioavailability of these Si products in more heterogeneous subjects, including women, and with a wider age range.

In conclusion, the present study reports that 3 different oral Si dietary supplements, in powder and liquid presentations, all provide highly bioavailable Si. Moreover, the absorption of Si from these 3 supplements, analysed by urinary Si excretion, was comparable between them, indicating an equivalent relative absorption of the analysed Si dietary supplements in healthy humans.

## Supplementary Information

Supplementary Figure S1.
