# Supplementary figures and images for: Relative absorption of silicon from different formulations of dietary supplements: a pilot randomized, double-blind, crossover post-prandial study
Source: Sci Rep. 2021 Aug 13;11:16479. doi: 10.1038/s41598-021-95220-2 (PMC8363645; doi:10.1038/s41598-021-95220-2)

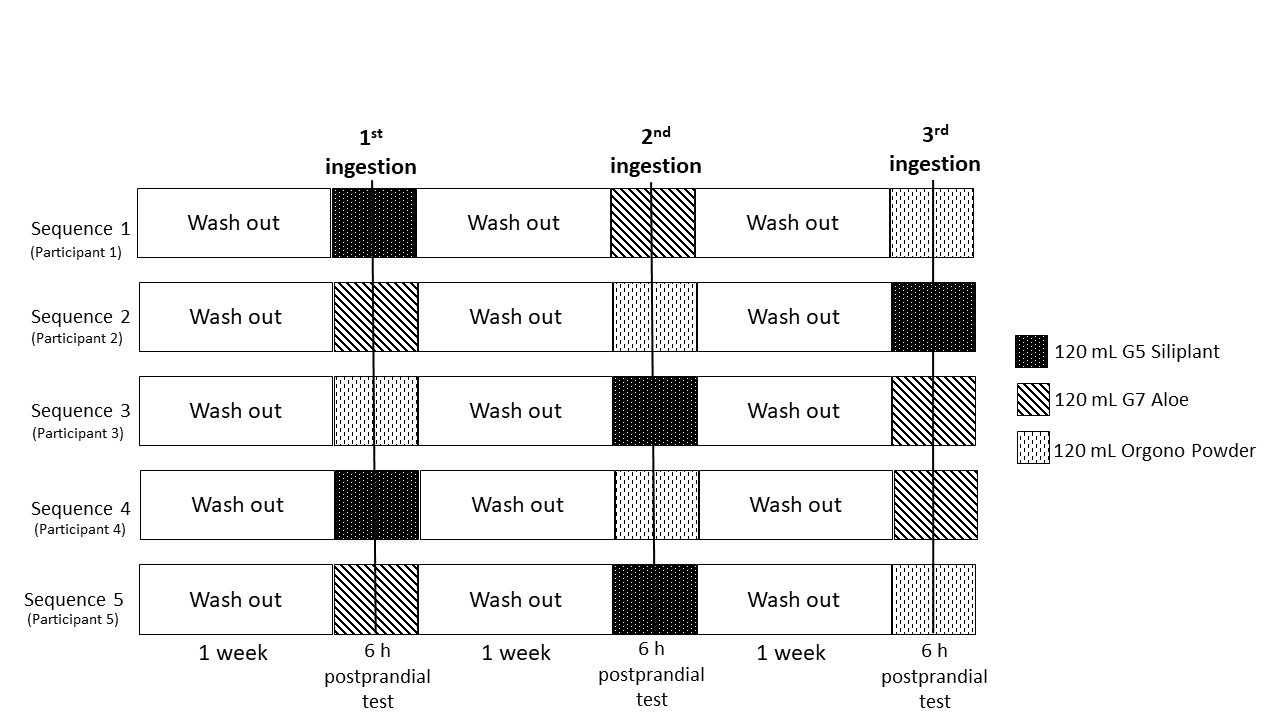

Supplement: Supplementary file 1 — Supplementary Figure S1. [file 41598_2021_95220_MOESM1_ESM.jpg]
